# Xanthine oxidoreductase activity is associated with serum uric acid and glycemic control in hemodialysis patients

**DOI:** 10.1038/s41598-017-15419-0

**Published:** 2017-11-13

**Authors:** Ayumi Nakatani, Shinya Nakatani, Eiji Ishimura, Takayo Murase, Takashi Nakamura, Mari Sakura, Yu Tateishi, Akihiro Tsuda, Masafumi Kurajoh, Katsuhito Mori, Masanori Emoto, Masaaki Inaba

**Affiliations:** 1 Departments of Metabolism, Endocrinology, and Molecular Medicine, Osaka City University Graduate School of Medicine, Osaka, Japan; 2 Departments of Nephrology, Osaka City University Graduate School of Medicine, Osaka, Japan; 3 Departments of Nephrology, Ishikiriseiki Hospital, Osaka, Japan; 4 Departments of Radioisotope and Chemical Analysis Center, Laboratory Management, Sanwa Kagaku Kenkyusho Co., Ltd, Nagoya, Aichi Japan; 5 Department Pharmacological Study Group, Pharmaceutical Research Laboratories, Sanwa Kagaku Kenkyusho Co., Ltd, Nagoya, Aichi Japan

## Abstract

Xanthine oxidoreductase activity (XOR-a) plays an important role as a pivotal source of reactive oxygen species. In the present study, we investigated factors associated with plasma XOR-a in 163 hemodialysis patients (age 67.3 ± 10.9 years; 89 males and 74 females), using a newly established, highly-sensitive assay based on [^13^C_2_,^15^N_2_] xanthine and liquid chromatography/triple quadrupole mass spectrometry. Plasma glucose and serum uric acid levels correlated significantly and positively with plasma XOR-a. In multiple regression analyses, the presence of type 2 diabetes mellitus (T2DM) and plasma glucose were associated significantly, independently, and positively with plasma XOR-a. While serum uric acid correlated significantly and positively with plasma XOR-a in hemodialysis patients without T2DM, plasma glucose and serum glycated albumin, a new marker of glycemic control in diabetic hemodialysis patients, correlated significantly and positively with plasma XOR-a in those with T2DM. Multivariate analyses in those with T2DM revealed that plasma glucose and serum glycated albumin were associated significantly and independently with plasma XOR-a, and that serum uric acid was associated significantly and independently with XOR-a in those without T2DM. Our results suggested that glycemic control in hemodialysis patients may be important in regard to a decrease in ROS induced by XOR.

## Introduction

Chronic kidney disease (CKD) and end-stage renal disease (ESRD) are strongly associated with cardiovascular disease (CVD)^1^. The high morbidity and mortality of CVD in CKD and ESRD patients cannot be explained by classical CVD risk factors alone, such as hypertension, smoking habits, and hypercholesterolemia^2^. Oxidative stress has been reported to be a novel, non-classical risk factor for CVD^3^. Xanthine oxidoreductase (XOR) is a ubiquitous enzyme that catalyzes the oxidation of both hypoxanthine to xanthine and xanthine to uric acid in the purine degradation pathway^4,5^. It has been reported that XOR plays an important role in a variety of physiological and pathophysiological conditions^6^, including endothelial dysfunction in patients with, CVD^7^ and ESRD^8^. Thus, it is clinically important to measure XOR activity in high-risk CVD populations, *i.e*., CKD and ESRD patients. However, there has been no previous investigation of the relationship between plasma XOR activity and clinical parameters in ESRD patients.

Recently we have developed a novel method to measure XOR activity in human plasma utilizing stable isotope-labeled [^13^C_2_,^15^N_2_] xanthine and liquid chromatography mass spectrometry, comprised of a Nano Space SI-2 LC system(LC/MS) and a TSQ-Quantum triple quadrupole mass spectrometer (TQMS). This assay provides highly accurate and highly sensitive measurements of human plasma XOR activity under physiologically equivalent conditions^9,10^. In the present study, we conducted a cross-sectional single center investigation of 163 hemodialysis patients, in which we measured plasma XOR activity using the newly developed method, and examined the relationship between plasma XOR activity and the clinical parameters.

## Results

### Clinical characteristics of the patients undergoing hemodialysis

**Figure 1:**
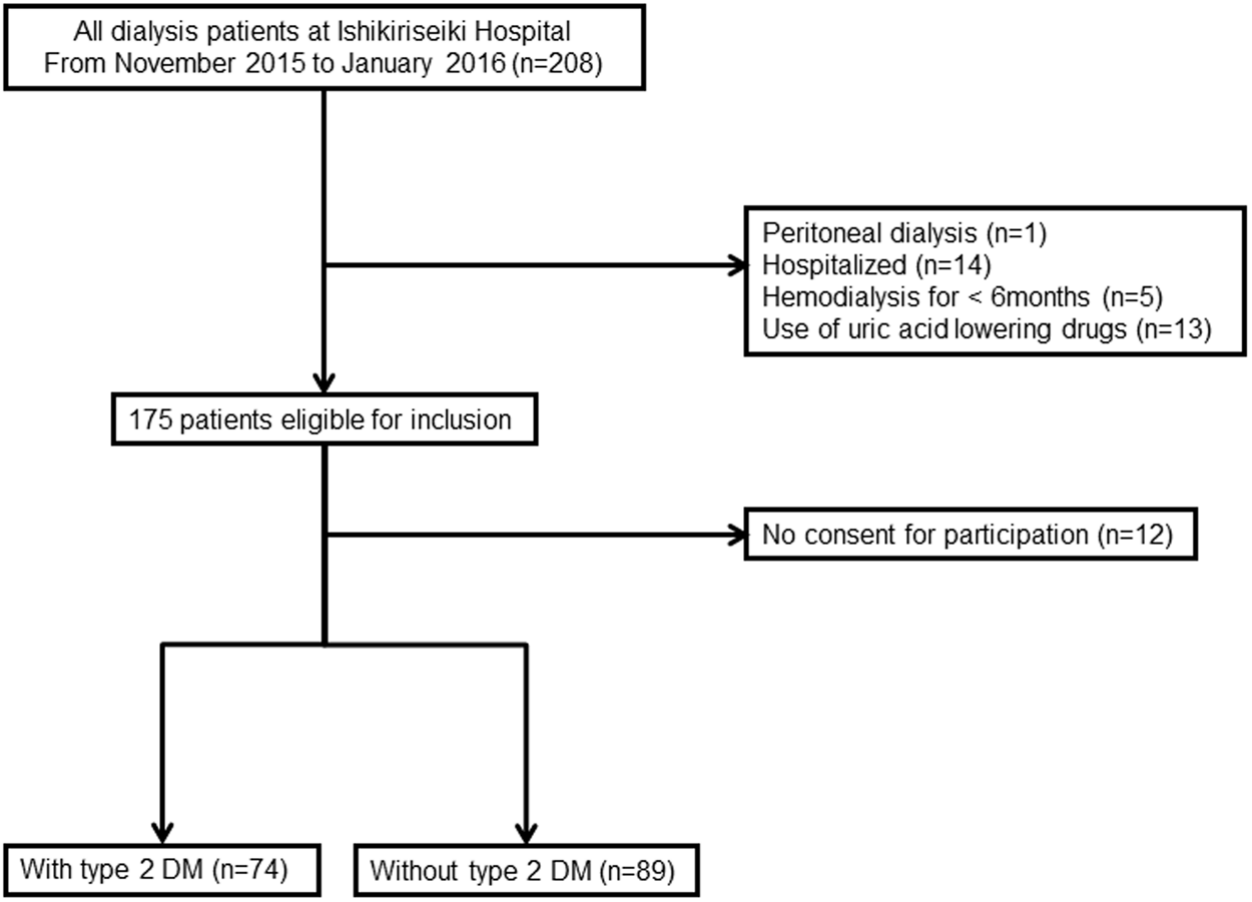
Flow of participants. This was a cross-sectional, single center study of maintenance hemodialysis patients. There were 74 and 89 patients with and without type 2 diabetes mellitus (DM), respectively.

Figure 1 presents a flow chart for the selection of participants in the study. Patients with peritoneal dialysis, hospitalization status, hemodialysis duration less than 6 months, or using uric acid lowering drug were excluded from the present study, thus 163 were enrolled. The clinical characteristics of all the hemodialysis patients, and those with and without type 2 DM are presented in Table 1. Plasma XOR activity in all hemodialysis patient was 21.4 ± 13.5 pmol/h/mL. Of importance, age (male 68.1 ± 10.2 *vs*. female 66.1 ± 11.9 years, p = 0.247) and serum uric acid level (male 7.3 ± 1.5 *vs*. female 7.3 ± 1.4 mg/dL, p = 0.900) were not significantly different between genders.

**Table 1:** Clinical characteristics of the hemodialysis patients and comparisons between those with and without type 2 diabetes mellitus (DM).

	All patients	Patients without type 2 DM	Patients with type 2 DM	*p*
Age (years)	67.3 ± 10.9	67.9 ± 11.1	66.8 ± 10.5	0.600
Gender (male/female)	163 (89/74)	89 (45/44)	74 (44/30)	0.256
Body mass index (kg/m^2^)	21.3 ± 3.95	20.9 ± 3.96	21.8 ± 3.96	0.07
Systolic blood pressure (mmHg)	149.4 ± 26.4	147.5 ± 24.7	151.7 ± 28.5	0.245
Diastolic blood pressure (mmHg)	75.3 ± 13.0	74.2 ± 9.27	76.7 ± 16.4	0.11
Dialysis duration (months)	100 ± 87	129 ± 100	66 ± 53	<**0.001**
Plasma glucose (mg/dL)	114 ± 44	97 ± 21	128 ± 43	<**0.001**
Glycated albumin (%)	—	—	20.1 ± 4.73	—
Diabetes duration (years)	—	—	22.9 ± 12.8	—
Urea nitrogen (mg/dL)	65 ± 16	66 ± 16	64 ± 16	0.381
Creatinine (mg/dL)	9.9 ± 2.4	10.3 ± 2.6	9.4 ± 2.1	**0.021**
Serum albumin (g/dL)	3.2 ± 0.3	3.2 ± 0.3	3.2 ± 0.3	0.859
Alanine transaminase (IU/L)	11 ± 7	11 ± 7	11 ± 7	0.932
Uric acid (mg/dL)	7.3 ± 1.5	7.4 ± 1.4	7.2 ± 1.5	0.184
Hemoglobin (g/dL)	10.5 ± 1.2	10.6 ± 1.3	10.4 ± 1.0	0.209
Hs-CRP^†^ (mg/dL)	0.15 ± 0.27	0.12 ± 0.12	0.19 ± 0.40	0.255
Corrected calcium (mg/dL)	9.5 ± 0.8	9.4 ± 0.8	9.5 ± 0.7	0.881
Phosphate (mg/dL)	5.4 ± 1.3	5.5 ± 1.3	5.4 ± 1.3	0.957
Alkaline phosphatase (IU/L)	278 ± 145	266 ± 143	285 ± 138	0.521
Intact-PTH^‡^ (pg/mL)	168 ± 132	169 ± 133	169 ± 133	0.954
XOR^§^ (pmol/h/mL)	21.4 ± 13.5	19.8 ± 12.4	23.4 ± 14.6	**0.032**

^†^Hs-CRP: high-sensitivity C-reactive protein, ^‡^PTH: parathyroid hormone, ^§^XOR: xanthine oxidoreductase.

Glycated albumin was only measured in hemodialysis patients with type 2 DM (n = 74).

*p < 0.05.

Data are expressed as the mean ± SD. Unpaired Student’s t-test was used for comparisons of continuous variables that exhibited a normal distribution. Mann-Whitney *U* test was used for comparisons of continuous variables with a skewed distribution. Chi-square test was used for comparisons of categorical variables.

### Correlations between plasma XOR activity and the clinical factors in all patients

The correlations between the clinical parameters and plasma XOR activity were examined by simple regression analyses. Uric acid, alanine transaminase, and plasma glucose were significantly and positively correlated with plasma XOR activity in all hemodialysis patients, though the correlations shown by r value were either weak or moderate (r = 0.228, p = 0.003; r = 0.445, p < 0.001; r = 0.229, p = 0.003, respectively) (Table 2).

**Table 2:** Correlations between the clinical parameters and plasma XOR^†^ activity in all patients (simple regression analyses) (n = 163).

	*r*	*p*
Age (years)	0.028	0.720
Dialysis duration (months)	−**0.150**	**0.045**
Serum albumin (g/dL)	0.017	0.833
Plasma glucose (mg/dL)	**0.229**	**0.003**
Urea nitrogen (mg/dL)	0.125	0.113
Creatinine (mg/dL)	−0.015	0.854
Alanine transaminase (IU/L)	**0.445**	<**0.001**
Uric acid (mg/dL)	**0.228**	**0.003**
Hemoglobin (g/dL)	0.120	0.128
Hs-CRP^‡^ (mg/L)	0.073	0.394
Corrected calcium (mg/dL)	−0.017	0.825
Phosphate (mg/dL)	0.102	0.196
Alkaline phosphatase (IU/L)	−0.031	0.690
Intact-PTH^§^ (pg/mL)	−0.121	0.125

^*^ *p* < 0.05. Data include the simple correlation coefficients (*r*) and the level of significance (*p*)

^†^XOR: xanthine oxidoreductase, ^‡^Hs-CRP: high-sensitivity C-reactive protein, ^§^PTH: parathyroid hormone.

### Multivariate analyses of factors associated with plasma XOR activity in all hemodialysis patients

In multiple regression analyses for plasma XOR activity (Table 3), the variables of age, gender, urea nitrogen, alanine transaminase, uric acid, and presence of DM were included in Model 1, and plasma glucose level was included instead of the presence of DM in Model 2, respectively, as explanatory variates. As shown in Table 3, alanine transaminase and the presence of DM were associated significantly and independently with plasma XOR activity in all hemodialysis patients (Table 3, Model 1) (alanine transaminase, β = 0.478, p < 0.001; presence of DM, β = 0.156, p = 0.028) (R^2^ = 0.262, p < 0.001). Alanine transaminase and plasma glucose levels were associated significantly and independently with plasma XOR activity in all hemodialysis patients (Table 3, Model 2) (alanine transaminase, β = 0.471, p < 0.001; plasma glucose levels, β = 0.300, p < 0.001, respectively) (R^2^ = 0.325, p < 0.001).

**Table 3:** Multiple regression analyses of plasma XOR^†^ activity in all hemodialysis patients.

	Model 1		Model 2	
	*β*	*p*	*β*	*p*
Age	0.033	0.632	0.002	0.978
Gender	0.103	0.146	0.124	0.067
Urea nitrogen	−0.016	0.832	−0.008	0.915
Alanine transaminase	**0.478**	<**0.001**	**0.471**	<**0.001**
Uric acid	0.105	0.178	0.073	0.324
Presence of DM^‡^	**0.156**	**0.028**	—	—
Plasma glucose	—	—	**0.300**	<**0.001**
R^2^	0.262 (p < 0.001)		0.325 (p < 0.001)	

^*^ *p* < 0.05. ^†^XOR: xanthine oxidoreductase, ^‡^DM: diabetes mellitus.

*β*: standardized correlation coefficient, R^2^: multiple coefficient of determination.

### Comparison of the clinical characteristics of hemodialysis patients with and without type 2 diabetes mellitus

Since the presence of DM and plasma glucose levels were associated significantly and independently with plasma XOR activity in all hemodialysis patients, in the next analysis, patients were divided into two groups; hemodialysis patients with and without type 2 DM. In hemodialysis patients with type 2 DM, diabetes duration and serum glycated albumin, a marker of glycemic control in patients receiving hemodialysis^11,12^, were 22.9 ± 12.8 years, and 20.1 ± 4.73%, respectively. As shown in Table 1, dialysis duration and serum creatinine levels were significantly shorter and lower, respectively, in patients with type 2 DM compared with those without. Plasma glucose levels were significantly elevated in hemodialysis patients with type 2 DM compared with those without (128 ± 43 *vs*. 97 ± 21 mg/L, p < 0.001). Other parameters, such as age, gender, serum albumin, urea nitrogen, alanine transaminase, uric acid, hemoglobin, high-sensitivity C-reactive protein, corrected calcium, phosphate, alkaline phosphatase, and intact PTH were not significantly different between the two groups.

Plasma XOR activity was significantly higher in hemodialysis patients with type 2 DM compared with those without (23.4 ± 14.6 *vs*. 19.8 ± 12.4 pmol/h/mL, p = 0.032) (Table 1).

### Correlations between plasma XOR activity and the clinical parameters in patients with and without type 2 diabetes mellitus

**Figure 2:**
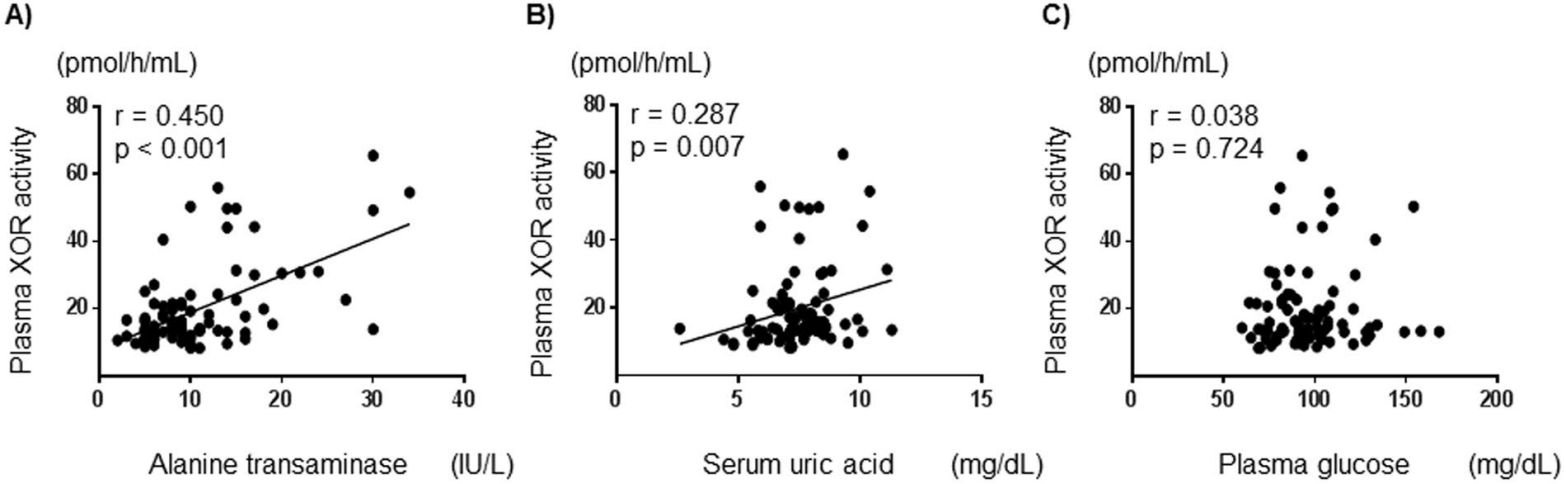
Correlations between plasma XOR activity and clinical parameters in hemodialysis patients without type 2 diabetes mellitus. Alanine transaminase (**A**), and uric acid (**B**) correlated significantly and positively with plasma XOR activity. Plasma glucose (**C**) did not correlate significantly with plasma XOR activity. XOR: xanthine oxidoreductase.

In hemodialysis patients without type 2 DM, alanine transaminase and uric acid levels correlated significantly and positively with plasma XOR activity (r = 0.450, p < 0.001; r = 0.287, p = 0.007, respectively) (Fig. 2A and B). In those patients, plasma glucose levels did not correlate significantly with plasma XOR activity (r = 0.038, p = 0.724) (Fig. 2C).

**Figure 3:**
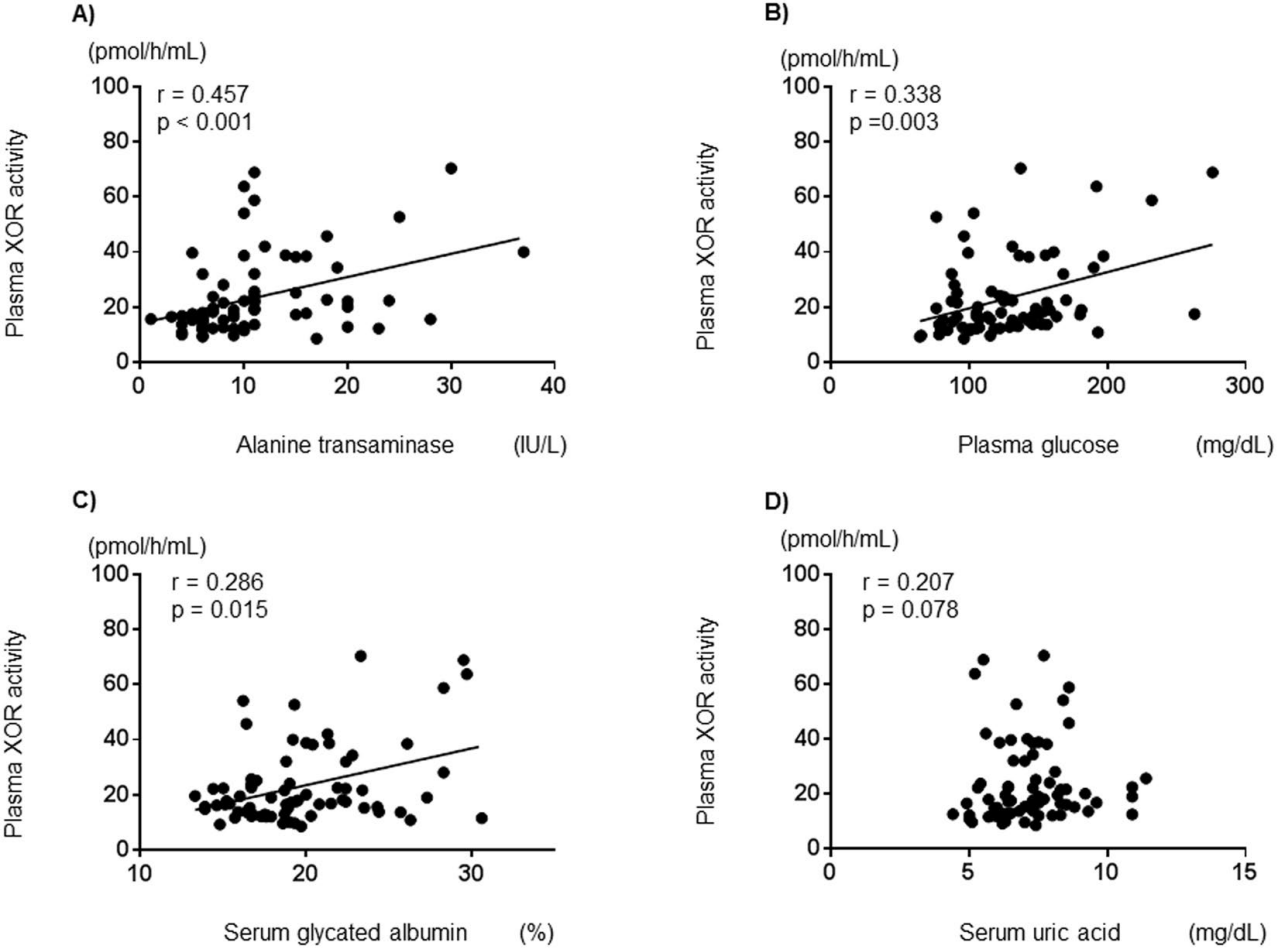
Correlations between plasma XOR activity and clinical parameters in hemodialysis patients with type 2 diabetes mellitus. Alanine transaminase (**A**), plasma glucose (**B**), and serum glycated albumin (**C**) correlated significantly and positively with plasma XOR activity. Serum uric acid (**D**) did not correlate significantly with plasma XOR activity. XOR: xanthine oxidoreductase.

In hemodialysis patients with type 2 DM, alanine transaminase, plasma glucose levels and serum glycated albumin, correlated significantly and positively with plasma XOR activity (r = 0.457, p < 0.001; r = 0.338, p = 0.003; r = 0.286, p = 0.015, respectively) (Fig. 3A,B and C). In those patients, serum uric acid levels did not correlate significantly with plasma XOR activity (r = 0.207, p = 0.078) (Fig. 3D).

### Multivariate analyses of factors associated with plasma XOR activity in patients with and without type 2 diabetes mellitus

As shown in Table 4, in hemodialysis patients without type 2 DM, alanine transaminase and serum uric acid levels were associated significantly and independently with plasma XOR activity (alanine transaminase, β = 0.562, p < 0.001; serum uric acid, β = 0.200, p = 0.042, respectively) (R^2^ = 0.395, p < 0.001). In those with type 2 DM, alanine transaminase and plasma glucose levels were associated significantly and independently with plasma XOR activity (Model 1) (alanine transaminase, β = 0.373, p = 0.001; and plasma glucose, β = 0.363, p = 0.001, respectively) (R^2^ = 0.301, p < 0.001). Alanine transaminase and glycated albumin were also associated significantly and independently with plasma XOR activity (Model 2) (alanine transaminase, β = 0.378, p = 0.001; glycated albumin, β = 0.330, p = 0.004, respectively) (R^2^ = 0.273, p = 0.003). Serum uric acid levels were not associated with plasma XOR activity in patients with type 2 DM.

**Table 4:** Multiple regression analysis of plasma XOR^†^ activity in hemodialysis patients with and without type 2diabetes mellitus (DM).

	Patients without type 2 DM		Patients with type 2 DM			
	*β*	*p*	Model 1		Model 2	
			*β*	*p*	*β*	*p*
Age	0.064	0.490	−0.026	0.810	0.001	0.999
Gender	0.128	0.150	0.083	0.453	0.012	0.917
Dialysis duration	−0.113	0.215	−0.057	0.598	−0.192	0.091
Urea nitrogen	−0.112	0.263	0.123	0.324	0.091	0.459
Alanine transaminase	**0.562**	<**0.001**	**0.373**	**0.001**	**0.378**	**0.001**
Uric acid	**0.200**	**0.042**	−0.063	0.594	−0.028	0.819
Plasma glucose	0.094	0.298	**0.363**	**0.001**	—	—
Glycated albumin	—	—	—	—	**0.330**	**0.004**
R^2^	0.395 (p < 0.001)		0.301 (p < 0.001)		0.273 (p = 0.003)	

^*^ *p* < 0.05, ^†^XOR: xanthine oxidoreductase.

*β*: standardized correlation coefficient, R^2^: multiple coefficient of determination.

## Discussion

In the present study, we investigated factors associated with plasma XOR activity in hemodialysis patients, using a newly established, highly-sensitive assay based on [^13^C_2_,^15^N_2_] xanthine and LC/TQMS. We demonstrated that plasma XOR activity was significantly higher in hemodialysis patients with type 2 DM, compared with those without.

Multivariate analysis revealed that, in hemodialysis patients without type 2 DM, serum uric acid level was associated significantly and independently with plasma XOR activity after adjustment for other confounders. In contrast, plasma glucose and glycated albumin, a new, better marker of the glycemic control index compared with hemoglobin A1c in diabetic hemodialysis patients^11,12^, was associated significantly and independently with plasma XOR activity in those with type 2 DM. Our findings suggest the importance of determining uric acid level in hemodialysis patients without type 2 DM and plasma glucose level in hemodialysis patients with type 2 DM in regard to plasma XOR activity.

Regarding DM, hepatic and plasma XOR activity have been reported to be 1.6- and 3.7-fold higher, respectively, in streptozotocin (STZ)-induced diabetic animals, compared with controls^13,14^. Our results in hemodialysis patients are considered to be consistent with those of the previous animal studies. Increased plasma XO activity has also been observed in patients with type 2 DM^15^. In addition, XO activity was previously found to be significantly and positively correlated with HbA1c in Asian patients with type 2 DM^16^. Although the mechanism remains unknown, redundant fructose in diabetic patients may increase XOR activity by increasing ATP degradation to AMP, a uric acid precursor^17^. Thus, in hemodialysis patients with type 2 DM, a high level of plasma glucose may be strongly associated with increased plasma XOR activity rather than uric acid level. In the present study, we demonstrated that plasma XOR activity was elevated significantly in hemodialysis patients with type 2 DM, compared with those without. We further demonstrated that plasma glucose levels and serum glycated albumin levels were associated significantly and independently with plasma XOR activity in these patients. Thus, glycemic control in hemodialysis patients may be important in regard to a decrease in ROS induced by XOR.

The results of the present study showed that, in stable maintenance hemodialysis patients, plasma XOR activity was significantly and independently associated with serum uric acid levels, and alanine transaminase; both of which are well known markers of elevated XOR^4,5,18^. Liver is the main sources of serum XO and hepatic damage caused by a variety of toxic agents has been reported to be associated with elevated serum XOR levels^4^. In the present patients, the serum level of alanine transaminase was less than 40 IU/L. Thus, even in patients with nearly normal liver function, plasma XOR activity was significantly and independently associated with serum alanine transaminase in all of the multiple regression analysis findings. Serum uric acid and alanine transaminase levels are affected by alcohol consumption. In the present patients, habitual alcohol drinking was only noted in 24 males and 9 females. Uric acid, alanine transaminase, and XOR levels in the alcohol drinkers and non-drinkers were 7.23 ± 1.84 *vs*. 7.31 ± 1.37 mg/dL, p = 0.819; 12.9 ± 8.31 *vs*. 10.4 ± 6.02 IU/L, p = 0.102; and 25.4 ± 18.9 *vs*. 20.4 ± 11.8 pmol/h/mL, p = 0.611, respectively.

In mammals, XOR exists in two interconvertible forms, *i.e*., xanthine dehydrogenase (XDH) and xanthine oxidase (XO)^19^. While XDH prefers NAD^+^ as an electron acceptor, XO transfers the electrons directly to molecular oxygen, resulting in the production of ROS^4,6,20^, *i.e*., superoxide anion and hydrogen peroxide, which have been implicated in the development of hypertension, dyslipidemia, and diabetes. ROS represent the main risk factor for atherosclerosis^4,20^. There have been few studies that have examined XOR, XO, and XDH levels in plasma from hemodialysis patients^8,21^. In a previous study, Boban M, *et al*. measured plasma levels of XOR, XO, and XDH in 28 patients with hemodialysis^8^. Although we were unable to simultaneously measure XO and XDH with our method, XO and XDH concentrations were consistently similar to those reported in their study. Thus, the plasma XOR (total amount of XO and XDH) levels obtained in the present study may have reflected plasma XO levels in our hemodialysis patients. Additional studies are needed to determine whether XOR and XO are useful markers for predicting CVD complications in patients receiving hemodialysis. Concerning the pattern of purine catabolism, Boban M, *et al*. also determined the levels of circulating purine compounds, such as triphosphate (ATP), adenosine diphosphate (ADP), and adenosine monophosphate (AMP). Although purine compound levels were not measured in the present hemodialysis patients, ATP concentration was significantly decreased, while ADP and ATP concentrations were significantly increased as compared to those in the control group of healthy individuals in their study^8^. The shift in pattern of nucleotide catabolism toward catabolic compounds may play an important role in increased XOR in hemodialysis patients. Further investigation is needed to determine the association between the pattern of nucleotide catabolism and increased XOR activity in hemodialysis patients including type 2DM.

Since the level of serum uric acid increases with age in women after menopause, multiple regression analyses were performed after dividing by gender. Those results showed that serum alanine transaminase and plasma glucose were significantly associated with plasma XOR activity in both male and female hemodialysis patients (Supplementary Table S1). However, the level of serum uric acid was not associated with plasma XOR activity in hemodialysis patients regardless of gender. Most female patients in the present study seemed to be post-menopausal, as the average age of menopause in Japan is approximately 50 years old^22,23^. Serum uric acid levels in the present males and females were 7.3 ± 1.5 and 7.3 ± 1.4 mg/dl, respectively (p = 0.900). We considered that the post-menopausal state of our female patients is the main factor for the similar results obtained in multiple-regression analyses after dividing by gender. Additional studies are needed to investigate the association between sex hormones and plasma XOR activity, especially in pre-menopausal younger women.

One advantage of the present study is presentation of findings obtained by measurement of human XOR activity with a newly established highly-sensitive assay that uses a combination of [^13^C_2_,^15^N_2_] xanthine and LC/TQMS. Traditionally, XOR activity assays have been based on determining the formation of uric acid from xanthine as a substrate with an ultraviolet detector^24,25^. However, methods that use such a protocol are not suitable for measuring uric acid-rich specimens such as human plasma, because subtraction of the basal level of uric acid originally contained in the sample is inevitable^26^. Such subtraction might have an adverse effect on measurement accuracy. To determine XOR activity in human plasma, Yamamoto *et al*. established an LC/fluorometric assay based on determination of isoxanthopterin formation from pterin (2-amino-4-hydroxypteridine)^27^. However, even though high sensitivity has been shown with that assay, the results are less representative of physiological condition as compared with an assay that uses xanthine, because pterin is not the primary substrate for XOR in mammals and its affinity for XOR is lower than that of xanthine^28^. Thus, we established a novel LC/TQMS assay based on measurement of stable isotope-labeled uric acid formation from isotope-labeled xanthine, which has been shown to have high sensitivity and requires no subtraction of the basal level^10^. In addition, the affinity of isotope-labeled xanthine for XOR is the same as that of xanthine^29^. This is the first study to measure human XOR activity in hemodialysis patients with this method, which we consider to provide information that is more directly representative of physiological condition than an assay that uses pterin.

There are some limitations to the present study. Firstly, the number of the subjects and patients examined in the present study was relatively small. This was mainly due to the fact that the study subjects were enrolled in a single institute. In addition, r values obtained in each of the correlation analyses were not high, possibly due to the limitations of this human clinical study, thus may not to be applicable. Secondly, in the present study, patients with hemodialysis duration less than 6 months were excluded, because we intended to study the metabolic condition of XOR in patients with a stable maintenance hemodialysis status (Fig. 1). However, residual renal function, as shown by urine output, is partially preserved in some patients. As for type 2 DM patients with residual renal function, serum uric acid level tends to be low, since they have increased urate excretion with glucose via URATv1^30,31^, which may have effects on high plasma XOR activity, particularly in type 2 DM patients. Lack of findings regarding residual renal function is one of the limitations of the present study. Thirdly, this study was cross-sectional one and did not demonstrate causality of the factors, *i.e*., poor glycemic control, increased uric acid, or hepatic dysfunction, that lead to increased plasma XOR activity. Further studies are required to explore whether plasma XOR activity can be reduced through strict uric acid control in hemodialysis patients without type 2 DM and by strict glycemic control in those with type 2 DM. Finally, the link between glycemic control and XOR activity should be confirmed in experimental animal models. *In vitro* studies may also be necessary to determine the potential effect of plasma glucose on XOR activity.

In conclusion, this is the first study to show that plasma XOR activity is associated significantly with serum uric acid levels in hemodialysis patients without type 2 DM, and that it is associated significantly with plasma glucose levels and serum glycated albumin levels in those with type 2 DM. Our results suggested that glycemic control in hemodialysis patients may be important in regard to a decrease in ROS induced by XOR.

## Material and Methods

### Ethics statement

The Institutional Review Board of Ishikiriseiki Hospital approved the use of the clinical data in accordance with the Declaration of Helsinki and the guidelines of Ishikiriseiki Hospital (approval no. 15–10). From November 2015 to February 2016, all participants in the present study provided written informed consent, both for the drawing of blood samples and for the use of data from their clinical records.

### Patients

All adult patients >18 years of age, who were treated with stable hemodialysis for at least 6 months, were included in this study. Patients were excluded if they did not provide informed consent to participate in the study. Thirteen patients, who had been treated with uric acid lowering drugs, such as allopurinol or febuxostat, were also excluded. Patients undergoing peritoneal dialysis, and those who were hospitalized were also excluded from the study. As for liver dysfunction, 2 patients with alanine transaminase greater than 40 IU/L were excluded, of whom 1 was admitted after the study had started and 1 had a hemodialysis duration of less than 6 months. Ultimately, we examined 163 hemodialysis patients (age 67.3 ± 10.9 years, 89 males and 74 females), which was comprised of 74 and 89 patients with and without type 2 DM, respectively. Residual renal function was none or very little in all hemodialysis patients.

### Blood sampling

Blood samples were obtained from the arteriovenous fistula just prior to the first hemodialysis session of the week. Routine laboratory tests were performed within 3 hours of blood sampling using an automated analyzer^11,32^. Glycated albumin was measured by an enzymatic method using the Lucica GA-L kit (Asahi Kasei Pharma Corp., Tokyo, Japan)^33^. Glycated albumin was hydrolyzed to amino acids by albumin-specific proteinase and then oxidized by ketoamine oxidase to produce hydrogen peroxide, which was measured quantitatively. The glycated albumin value was calculated as the percentage of glycated albumin relative to total albumin, which was measured using a new bromocresol purple method with the same serum sample^33^. Plasma glucose levels in hemodialysis patients in this study were obtained during random blood testing. Serum calcium concentrations were corrected to a serum albumin level of 4.0 g/dL according to a previously published formula^34^, as follows: corrected Ca (mg/dL) = [4.0 − albumin (g/dL)] + Ca (mg/dL).

### Measurement of plasma XOR activity

Measurement of plasma XOR activity was performed using freshly frozen samples that were maintained at −80 °C until the time of assay. Plasma XOR activity was measured using the recently-established assay using stable isotope-labeled [^13^C_2_,^15^N_2_] xanthine with liquid chromatography mass spectrometry (Nano Space SI-2 LC system(LC/MS), Shiseido, Tokyo, Japan, and a TSQ-Quantum triple quadrupole mass spectrometer (TQMS), Thermo Fisher Scientific GmbH, Bremen, Germany), as described previously^9,10^. In brief, 100 µL aliquots of plasma (purified on Sephadex G25 columns) were mixed with Tris buffer (pH 8.5) containing [^13^C_2_,^15^N_2_] xanthine as substrate, NAD^+^, and [^13^C_3_,^15^N_3_] uric acid as an internal standard. The mixtures were incubated at 37 °C for 90 min. The mixtures were subsequently combined with methanol (500 µL) and centrifuged at 2,000 *g* for 15 min at 4 °C. The supernatants were transferred to new tubes and dried using a centrifugal evaporator. The residues were reconstituted in 150 μL of distilled water, filtered through an ultrafiltration membrane, and measured using LC/TQMS. Calibration standard samples were measured for [^13^C_2_,^15^N_2_] uric acid, and the amounts of [^13^C_2_,^15^N_2_] uric acid produced were calculated from the calibration curve. XOR activities were expressed as [^13^C_2_,^15^N_2_] uric acid produced in pmol/mL/h. The intra- and inter-assay coefficients of variation were 6.5% and 9.1%, respectively^9^.

### Statistical analyses

Statistical analyses were performed using Graphpad Prism version 6.0 for Windows (Graphpad Software, San Diego, CA) and JMP software version 10 (SAS Institute Inc., Cary, NC). Values are expressed as the mean ± SD. Comparisons between hemodialysis patients with and without type 2 DM were made using unpaired Student’s t-test or Mann–Whitney *U* test for continuous variables, and chi-square test for categorical variables. Correlations between XOR activity and the clinical data were examined by Pearson’s and Spearman’s analyses for parametric and nonparametric data, respectively. Independent associations between the variables and XOR activity in hemodialysis patients were assessed by multiple regression analyses. P-values < 0.05 were considered statistically significant. The datasets generated during and/or analysed during the current study are available from the corresponding author on reasonable request.

## Electronic supplementary material

Supplementary Table S1
