# Supplementary material for: Xanthine oxidoreductase activity is associated with serum uric acid and glycemic control in hemodialysis patients
Source: Sci Rep. 2017 Nov 13;7:15416. doi: 10.1038/s41598-017-15419-0 (PMC5684129; doi:10.1038/s41598-017-15419-0)
Supplement: Supplementary file 1 — Supplementary Table S1 [file 41598_2017_15419_MOESM1_ESM.doc]

Xanthine oxidoreductase activity is associated with serum uric acid and glycemic control in hemodialysis patients

Ayumi Nakatani ^1)^, Shinya Nakatani ^1, 2, 3)^, Eiji Ishimura *^2)^, Takayo Murase ^4)^, Takashi Nakamura ^5)^, Mari Sakura ^3)^, Yu Tateishi ^3)^, Akihiro Tsuda ^1)^, Masafumi Kurajo ^1)^, Katsuhito Mori ^2)^, Masanori Emoto ^1)^, and Masaaki Inaba ^1, 2)^

AN, SN, and EI contributed equally to this study as first author.

^1)^ Departments of Metabolism, Endocrinology, and Molecular Medicine, Osaka City University Graduate School of Medicine, Osaka, Japan

^2)^ Departments of Nephrology, Osaka City University Graduate School of Medicine, Osaka, Japan

^3)^ Departments of Nephrology, Ishikiriseiki Hospital, Osaka Japan

^4)^ Departments of Radioisotope and Chemical Analysis Center, Laboratory Management, Sanwa Kagaku

Kenkyusho Co., Ltd., Nagoya, Aichi, Japan

^5)^ Department Pharmacological Study Group, Pharmaceutical Research Laboratories, Sanwa Kagaku Kenkyusho Co., Ltd., Nagoya, Aichi, Japan

***Word coun*t:** Title, 20 words; Abstract, 197 words; Main body 3481 words

***Short Title*:** XOR activity in hemodialysis patients

***Key words*:** xanthine oxidoreductase activity, hemodialysis, uric acid, type 2 diabetes mellitus

***Corresponding author and address of reprint request*:**

Eiji Ishimura, MD, PhD, FASN, FACP

Departments of Nephrology, Osaka City University Graduate School of Medicine

1-4-3, Asahi-machi, Abeno-ku, Osaka 545-8585, Japan

TEL: +81-6-6645-3806, FAX: +81-6-6645-3808, e-mail: [ish@med.osaka-cu.ac.jp](mailto:ish@med.osaka-cu.ac.jp)

Supplementary Table S1. Multiple regression analyses of plasma XOR^†^ activity in hemodialysis patients of male and female

|  | Male | | Female | |
| --- | --- | --- | --- | --- |
|  | *β* | *p* | *β* | *p* |
| Age | -0.075 | 0.402 | 0.148 | 0.140 |
| Urea nitrogen | 0.001 | 0.994 | 0.056 | 0.645 |
| Alanine transaminase | **0.409** | **<0.001** | **0.622** | **<0.001** |
| Uric acid | 0.121 | 0.215 | -0.080 | 0.508 |
| Plasma glucose | **0.327** | **<0.001** | **0.240** | **0.018** |
| R^2^ | 0.318  (p <0.001) | | 0.420  (p < 0.001) | |

**p*< 0.05. †XOR: xanthine oxidoreductase.

*β*: standardized correlation coefficient, R^2^: multiple coefficient of determination.
